# Evaluation of the Swedish breeding program for cavalier King Charles spaniels

**DOI:** 10.1186/1751-0147-52-54

**Published:** 2010-09-23

**Authors:** Tobias Lundin, Clarence Kvart

**Affiliations:** 1 Veterinary Animal Hospital Smådjursakuten, Ingeborgsgatan 6, 416 59 Gothenburg, Sweden; 2 Dept of Anatomy, Physiology and Biochemistry, Swedish University of Agricultural Sciences, 750 07 Uppsala, Sweden

## Abstract

A breeding program with the aim of reducing the prevalence of mitral regurgitation (MR) caused by myxomatous mitral valve disease (MMVD) in Cavalier King Charles Spaniels (CKCS) is currently ongoing in Sweden. In this investigation 353 CKCS were selected as a sample of the population and 150 were examined by auscultation for heart murmurs when they reached the age of six years in 2007 and 2009. The aim with this investigation was to study the prevalence of heart murmurs in six-year-old CKCS and to estimate if prevalence has decreased since the breeding program was introduced 2001. The effect of the breeding program was evaluated by comparing the prevalence of heart murmurs in the two groups. In 2007, the prevalence of heart murmurs was 52% (50% for females and 54% for males) and in 2009, the prevalence was 55% (44% for females and 67% for males). No significant difference was found in the prevalence of heart murmurs between 2007 and 2009 (P = 0.8). For all six-year-old CKCS, the prevalence of heart murmur was 53% (females 46% and males 61%), which is higher than previous Swedish investigations.

## Background

Mitral regurgitation (MR), secondary to myxomatous mitral valve disease (MMVD), is the most common cardiac disorder in dogs and is usually caused by progressive degeneration of the atrioventricular valves. Degeneration of the atrioventricular valves renders the leaflets thicker and irregular, leading to insufficient coaptation of the leaflets and regurgitation of blood with accompanying enlargement of the atria and ventricles. These pathological changes are associated with a characteristic systolic heart murmur when the valves become incompetent and blood is ejected back into the atrium during systole. The mitral valve alone or both the mitral and tricuspid valves may be affected: the tricuspid valve alone, aortic, or pulmonary valves are less commonly affected. The dog can compensate for valvular insufficiency for a period, but with progression of valve degeneration, usually left side, congestive heart failure ultimately develops. MMVD is found in all dogs but is more common in small to medium-size breeds, such as Poodle, Papillon, Dachshund, Chihuahua and CKCS [1,2]. In CKCS onset is early with a high prevalence of MR caused by MMVD, and at the age of 6-7 years, murmur prevalence is approximately 50%. At the age of 11 years, almost 100% of CKCS have developed MMVD [3-8].

MMVD is considered highly inheritable with a polygenic threshold, which means that multiple genes influence the trait and a certain threshold has to be reached before MMVD and MR develops [9,10]. Males have a lower threshold than females, meaning in a population of dogs with the same genotype, male dogs will develop MMVD at a lower age than females. If two dogs with late onset MMVD mate, the offspring will, on average, have late onset MMVD, and vice versa [10].

Based on this knowledge, the Swedish Kennel club and the Special club for cavalier King Charles spaniels (SCKCS) started a breeding program in 2001 with the aim of reducing MMVD in the Swedish population of CKCS. In this program, dogs are not allowed to breed until four years of age and need a heart auscultation without murmurs within eight months before mating. However, dogs are allowed to breed at an age of 24 months, if the dog and its parents are examined and no murmurs are detected. Male dogs that have a heart auscultation at seven years of age without murmurs are allowed to breed without further heart evaluation. Breeding animals whose parents have heart murmurs before four years of age are not allowed to breed [11]. The aim of this investigation was to study the prevalence of heart murmurs in the Swedish population of six-year-old CKCS born 2001 and 2003, and to estimate if prevalence has decreased since the breeding program was introduced 2001.

## Materials and methods

### Material

**Figure 1:**
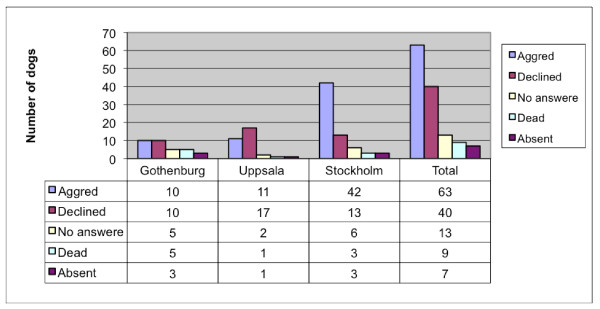
**Answers from letters requesting agreement for cardiac auscultation in dogs born 2001**.

**Figure 2:**
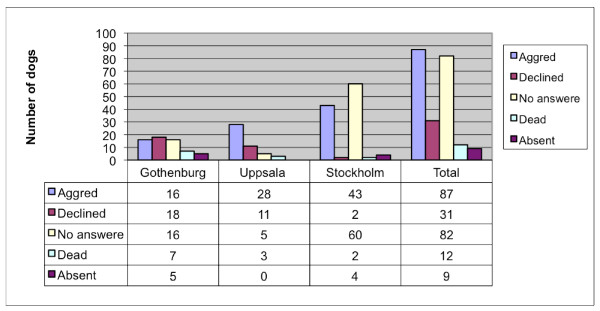
**Response to letters requesting agreement for cardiac auscultation in dogs born 2003**.

Six-years-old CKCS (n = 150) breed according to the rules of the Swedish breeding program were examined with cardiac auscultation to detect the prevalence of heart murmurs. All CKCS born in 2001 (n = 132) and 2003 (n = 221), registered within the Swedish kennel club, and living in a radius of 150 km of Uppsala, Gothenburg, or Stockholm were listed. The owners of the dogs received a letter asking for agreement to cardiac auscultation of their dog/s. The responses to the letter are summarized in Figures 1 and 2.

In the 2001 group, 63 dogs were examined, of which seven were excluded after physical examination: two dogs because of the wrong site of the heart murmur (pulmonic) and, five because they been bred after parents not approved by the breeding program. The dogs were examined between May and November 2007, resulting in 56 approved CKCS (30 females and 26 males) with a mean age of 6.2 years (range from 5.6 to 6.6 years).

In the 2003 group, 87 dogs were examined, 12 dogs were excluded after examination for not being bred by parents approved by the breeding program. The dogs were examined between April and May 2009, resulting in 75 CKCS (39 females and 36 males) with a mean age of 5.9 years (range from 5.2 to 6.3 years). The prevalence and grade of heart murmur, geographical location and, gender were recorded for the 131 dogs.

The prevalence of heart murmurs in the Swedish population of six-year-old CKCS born 2001 and 2003 were calculated from all the 131 approved dogs. To estimate if the prevalence is decreasing, since the breeding program was introduced 2001, comparison were made between the prevalence and intensity of heart murmurs between 2001 and 2003.

The family relations in each group were investigated through comparison of the dog's parents in each group. In the 2001 group, 37 dogs had one or more littermate and there were 14 siblings with one common parent, seven of these had siblings with the same parents and one sibling with one common parent. In the 2003 group, 30 dogs had one or more littermate and there were 40 siblings with one common parent, 18 of these had several siblings with the same parents and one sibling with one common parent.

### Auscultation

All dogs were examined standing on a table in a quiet room after acclimatizing to the environment for three minutes. The auscultations were performed by the same examiner (T Lundin) with a Littman Classic II S.E stethoscope. The examiner's ability to detect and grade heart murmurs was evaluated by an experienced veterinary cardiologist (C Kvart) and found to be accurate. The existence of cardiac murmur, intensity (grade 1-6) and, site were recorded according to Gompf (1988) [12]. Auscultation and grading of heart murmurs is a subjective method, and to compensate for this, the two groups of dogs were divided in to four groups:

Without murmur.

Low-intensity murmur, grades 1 to 2 according to Gompf (1988).

Moderate-intensity murmur, grades 3 to 4 according to Gompf (1988).

High-intensity murmur, grades 5 to 6 according to Gompf (1988).

All statistics were calculated from these eight groups.

### Statistical methods

All statistics were calculated by a commercially available statistics program (JMP V.5.0, SAS Inc, Cary, NC, USA.). The statistical method used for comparison between categorical data was chi^2 ^test and comparison for continuous data was with student's t-test. To evaluate the effect of gender and site of auscultation multi regression analyzes were used. The minimum level of significance was chosen as P < 0.05.

## Results

### Dogs born in 2001

**Figure 3:**
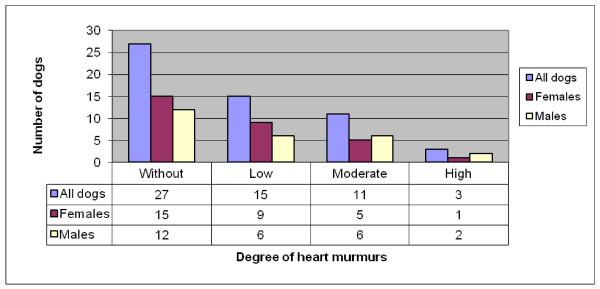
**Distribution of heart murmurs by murmur grade and gender in 56 CKCS born 2001 (30 females and 26 males)**.

Twenty-nine (52%) of the 56 CKCS had some degree of heart murmur, 15 (50%) of the 30 females and 14 (54%) of the 26 males had some degree of heart murmur. The distribution of grade of heart murmurs is summarized in Figure 3.

No statistical differences were found in the prevalence of heart murmurs between female and male dogs (P = 0.8) or murmur grade between female and male dogs (P = 0.67).

### Dogs born in 2003

**Figure 4:**
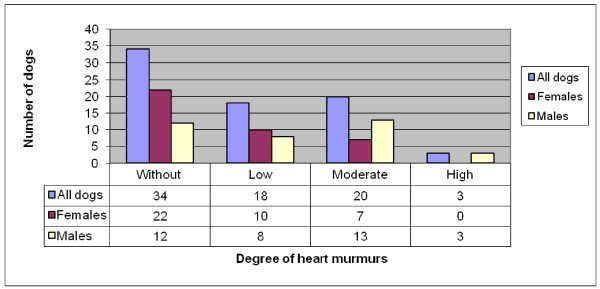
**Distribution of heart murmurs by murmur grade and gender in 75 CKCS born 2003 (39 females and 36 males)**.

Forty-one (55%) of the 75 CKCS had some degree of heart murmur, 17 (44%) of the 39 females and 24 (67%) of the 36 males had some degree of heart murmur. The distribution of grade of heart murmurs is summarized in Figure 4.

No statistical difference was found in the prevalence of heart murmurs between male and female dogs (P = 0.05). Male dogs had a higher grade of heart murmurs than female dogs (P = 0.02).

### Dogs born in 2001 and 2003

**Figure 5:**
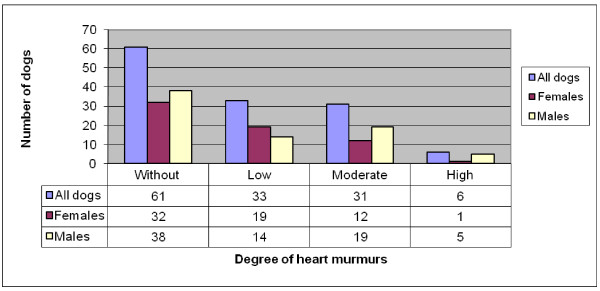
**Distribution of heart murmurs by murmur grade and gender in all CKCS born 2001 and 2003 (69 females and 62 males)**.

Seventy (53%) of the 131 dogs had some degree of heart murmur, 32 (46%) of the 69 females and 38 (61%) of the 62 males had some degree of heart murmur. The distribution of grade of heart murmurs is summarized in Figure 5.

No statistical difference was found in the prevalence of heart murmurs between females and males (P = 0.08). Male dogs had greater intensity of heart murmurs than female dogs (P = 0.04).

### Comparison between dogs born in 2001 and 2003

No statistical difference was found in the prevalence of heart murmurs (P = 0.8) or in the intensity of heart murmurs (P = 0.8) between dogs born in 2001 and dogs born in 2003. There was no statistical difference in the prevalence (P = 1) of heart murmurs or the intensity (P = 0.5) of heart murmurs between the geographical locations of the dogs nor between the age of the two groups, the prevalence of females and males or, between the age of females and males between the two groups.

## Discussion

This study revealed a high prevalence of heart murmurs (52-55%) in six-year-old CKCS. Furthermore, there was no improvement in the proportion of dogs with heart murmurs or grade of heart murmurs between the CKCS born 2001 and those born in 2003. During this period, the breeding program aimed at reducing the prevalence of MMVD in Swedish CKCS was active. The prevalence of murmurs and indirect MMVD among the six-year-old CKCS was 52% in 2007 and 55% in 2009, which corresponded to findings in earlier studies [3-8]. Before the breeding program was introduced in Sweden, the prevalence of MMVD in six-year-old CKCS was 35.5% (27% for females and 44% for males), based on a previous study between 1985-1991 [5]. In the present investigation, 53% of all dogs had heart murmurs, with 46% of the females affected and 61% of the males. However, this should not be interpreted as an increased prevalence of heart murmurs, as the demographics of the populations of dogs differed from the dogs examined in 1985-1991 and different observers examined the dogs. In the study from 1985-1991, the age range was larger with 64% of the dogs being less than three years of age and only a few (n = 20) six years, as most dogs were examined at dog shows [5]. This presumably led to an underestimation of the prevalence of MMVD in the Swedish population of six-year-old CKCS between 1985 and 1991. In the present investigation, the prevalence of MMVD was evaluated through comparison between two samples of six-year-old CKCS in similar geographical locations. With 131 dogs included in the investigation, this result is probably closer to the true prevalence of MMVD within the Swedish population of six-year-old CKCS. Despite the high levels of 52% in 2001 and 55% in 2003, there is a risk these values were underestimated, as owners of dogs in which MMVD already been detected may have been less motivated to participate in the study.

The breeding program has been evaluated through comparison between the two populations of dogs, which requires the groups are comparable. The dogs were selected from the same areas and in equal manner to minimize divergence between the groups. The prevalence of MMVD in CKCS increases with age and male dogs develop MMVD earlier than females, thus, an age difference between the groups or more males in one groups should affect the result [5]. However, no statistical difference was determined between the age and prevalence of gender between the two groups investigated. In the group of dogs born in 2001, there was no statistical difference in the intensity of heart murmurs between females and males, but in the group of dogs born 2003, and for all dogs, male dogs with heart murmurs had murmurs of greater intensity than females with heart murmurs: this result was in agreement with findings from earlier studies [10]. As less dogs were born in 2001 when the breeding program was introduced, this could be one explanation for the limited number of dogs and lack of differences in the findings in the group from 2001. Mitral systolic heart murmur is not a definitive diagnosis for MMVD in CKCS [13,14]. However, in the breeding program against MMVD, auscultation of mitral systolic heart murmur is the screening method used for the disease, as other causes for this form of murmur, in middle age or older CKCS, are extremely rare [7]. Therefore, this method was also used in this investigation and to minimize variation, the same person examined all dogs with the same stethoscope. Family relations within the samples of dogs were a factor that could affect the prevalence found in 2007 and 2009; however, the family relations within the two samples were similar. In the breeding program, dogs are not allowed to breed until the age of four years unless the parents were free of heart murmurs at four years of age, then the dog are allowed to breed from two years of age [11]. Based on the prevalence of MMVD from 1985-1991 this would exclude 13%, 12% for females and 15% for males, of the CKCS from breeding each year, still less than 50% which is important for not creating other genetic problems [5].

This study investigated the prevalence in six-year-old CKCS and the difference in prevalence and grade of heart murmur between two groups of six-year-old dogs with two years between. No improvement on the prevalence of the disease was detected during this period, indicating the breeding program does not have the desired effect. This result corroborates statistics from Agria pet insurance company, which also indicate there is lack of improvement regarding death from MMVD (Brenda Bonnett personal communication 2009). However, the breeding program might have a slow effect on disease prevalence over a longer period, especially in dogs less than four years of age.

The underlying causes for the findings of the present study are unclear. Possible explanations include several factors such as a too low age limit for breeding, import of breeding dogs with unknown ancestral background, a lower inheritance of MMVD than previously estimated, inadequate compliance to the breeding programs among breeders, and insensitive screening methods. Furthermore, the screening program only encourages breeders to screen dogs up to a certain age and many dogs develop MMVD after that age. Therefore, continued screening of dogs used for breeding until they develop a heart murmur should be beneficial for obtaining a complete view of the onset of heart murmurs within the breed and facilitate breeding against MMVD. This knowledge is of direct relevance for any organization considering breeding programs against MMVD, and not at least for the Swedish Kennel club and the SCKCS as a stimulus to reform the ongoing breeding program.

## Conclusions

The result from this investigation indicates that the prevalence of MMVD in six-year-old cavalier King Charles spaniels, born 2001 and 2003, is at least 50% and lacks signs of decrease despite the current breeding program introduced in Sweden 2001.

## Competing interests

The authors declare that they have no competing interests.

## Authors' contributions

Both authors have participated in the main part of the work: Planning of the study, auscultation of dogs (initially both investigators and the later part by TL), preparation of manuscript. Both authors read and approved the final manuscript.
